# Monod’s sign in a rare case of aspergilloma

**DOI:** 10.11604/pamj.2023.45.176.41234

**Published:** 2023-08-22

**Authors:** Souvik Sarkar, Babaji Ghewade

**Affiliations:** 1 Department of Respiratory Medicine, Datta Meghe Institute of Higher Education and Research, Wardha, Maharashtra, India

**Keywords:** Pulmonary aspergilloma, monod’s sign, aspergillus fumigatus

## Image in medicine

A fifty-year-old male came to the casualty with fever, chills, blood in cough, breathlessness, and right-sided chest pain for 2 months. He had a few episodes of hemoptysis approximately 5-10 ml per episode, of bright red colour. The patient was a chronic alcoholic and also had a history of tuberculosis 11 years back for which he took treatment of 6 months. On examination, he was thin built, febrile, had a pulse rate of 116 beats per minute, a blood pressure of 100/60 mmHg. On respiratory system examination a bronchial breath sound was heard in the right mammary region. A chest X-ray was done of the patient which showed fibro-bronchiectatic changes in the right upper zone. The patient also underwent high resolution computed tomography of the lungs which showed a round enhancing lesion with a surrounding air shadow seen in the superior basal segment of the right lower lobe measuring 2.5 cm x 1.6 cm in size which also changed in orientation with a change in position of the patient; Monod´s sign. Typical Monod´s sign along with air crescent sign. The patient´s serum IgE antibody for *Aspergillus fumigatus* was positive, and thus a diagnosis of fungal ball or aspergilloma was confirmed. The patient was started on oral itraconazole, oral tranexamic acid which controlled the hemoptysis in a week's duration and was then referred to a thoracic surgeon for pneumonectomy.

**Figure 1:**
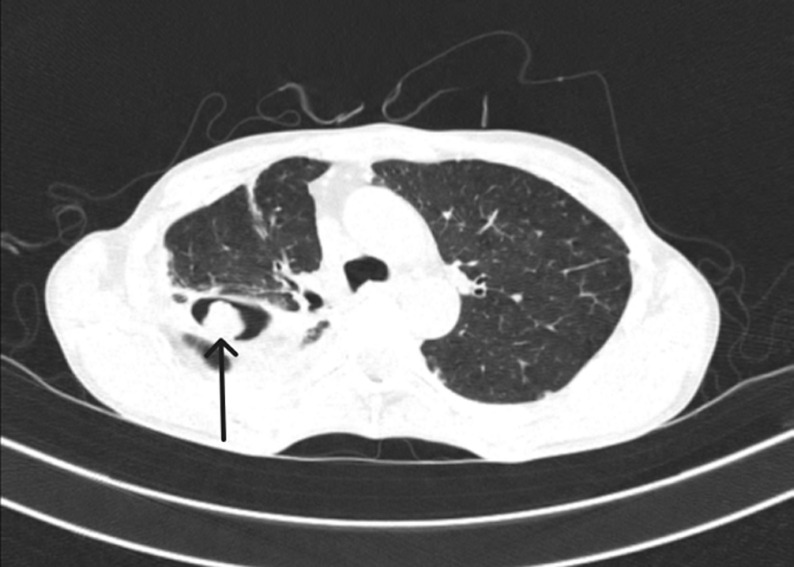
an axial section of computed tomography of lung showing a round enhancing lesion with a surrounding air shadow seen in the superior basal segment of the right lower lobe measuring 2.5 cm x 1.6 cm in size which also changed in orientation with a change in position of the patient; Monod's sign

